# Epidemiology and assessments of delirium in nursing homes and rehabilitation facilities: a cross-country perspective

**DOI:** 10.1007/s41999-025-01207-x

**Published:** 2025-04-28

**Authors:** Alice M. Ornago, Elena Pinardi, Maria Cristina Ferrara, Suzanne Timmons, Chukwuma Okoye, Alberto Finazzi, Paolo Mazzola, Peter Nydahl, Rebecca von Haken, Heidi Lindroth, Keibun Liu, Alessandro Morandi, Giuseppe Bellelli

**Affiliations:** 1 School of Medicine and Surgery, University of Milano-Bicocca, Piazza dell’Ateneo Nuovo 1, Milan, Italy; 2 Aging Research Center, Department of Neurobiology, Care Sciences and Society, Karolinska Institutet and Stockholm University, Stockholm, Sweden; 3 Mercy University Hospital and St. Finbarr’s Hospital, Cork, Ireland; 4 Acute Geriatric Unit, IRCCS San Gerardo Foundation, Monza, Italy; 5 Nursing Research, University Hospital Schleswig-Holstein, Kiel, Germany; 6 Institute of Nursing Science and Development, Paracelsus Medical University, Salzburg, Austria; 7 Department of Surgery, University Hospital Mannheim, Mannheim, Germany; 8 Division of Nursing Research, Department of Nursing, Mayo Clinic, Rochester, MN USA; 9 Center for Aging Research, Regenstrief Institute, Center for Health Innovation and Implementation Science, School of Medicine, Indiana University, Indianapolis, IN USA; 10 ICU Collaboration Network (ICON), Bunkyo-Ku, Tokyo Japan; 11 Intermediate Care and Rehabilitation, Azienda Speciale Cremona Solidale, Cremona, Italy; 12 University of Brescia, Brescia, Italy

**Keywords:** Delirium, Long-term care, Nursing homes, Rehabilitation, Epidemiology

## Abstract

**Purpose:**

Delirium represents a significant healthcare challenge in long-term care settings. This study aims to investigate the epidemiology of delirium in long-term care facilities (LTCFs) across various countries, examining point prevalence and assessment methods.

**Methods:**

This descriptive analysis included data from 94 surveys conducted in LTCFs on World Delirium Awareness Day (WDAD), March 15th, 2023. Group comparisons were made between rehabilitation facilities (RFs) and nursing homes (NHs).

**Results:**

Of the participating units/wards, primarily from Europe and Australia, 65 were RFs and 29 were NHs. The overall reported delirium point prevalence was approximately 12%, with higher rates in RFs than in NHs. While most units/wards reported using a validated delirium detection tool, notable differences emerged in the frequency of assessments, the personnel conducting them, and delirium awareness interventions across settings.

**Conclusion:**

Despite the well-documented impact of delirium on residents, the international variability in clinical practices within LTCFs highlights the current inadequacy in adopting effective and consistent strategies to address this condition.

**Supplementary Information:**

The online version contains supplementary material available at 10.1007/s41999-025-01207-x.

## Key summary points

**Aim:**

To explore delirium prevalence and assessment methods in long-term care facilities across multiple countries.

**Findings:**

A reported delirium point prevalence of approximately 12% was found, with higher rates in rehabilitation facilities compared to nursing homes, along with significant variability in screening and assessment practices.

**Message:**

Implementing standardized and objective approaches to delirium identification is crucially needed in long-term care settings worldwide.

**Supplementary Information:**

The online version contains supplementary material available at 10.1007/s41999-025-01207-x.

## Introduction

Delirium is a neuropsychiatric syndrome characterized by the rapid onset of fluctuating attention, disorganized thinking, and altered levels of consciousness, following acute medical conditions [1]. It represents a significant healthcare challenge, particularly within long-term care (LTC) settings, where its impact is heightened by the vulnerability of the resident population. A recent study by Webber et al., involving 92,005 older adults in LTC across Canada, showed that delirium significantly affected both patients’ prognosis and cognitive trajectory at a one-year follow-up [2]. In addition, delirium in LTC residents has been associated with increased risk of falls, more rapid functional decline, frequent hospital admissions, and higher healthcare costs [3].

Since LTC services are designed to assist individuals who experience diminished functional ability or need ongoing nursing care [4, 5], the beneficiaries are predominantly older, with a significant proportion of those aged 80 and above both in Europe and North America [6, 7]. Moreover, they are often characterized by various degrees of frailty and cognitive impairment, all of which are well-established predisposing factors for delirium onset [8, 9]. However, the prevalence of delirium in LTC settings varies widely across the literature, possibly reflecting heterogeneity in diagnostic resources, local healthcare practices, and resident demographics. A review encompassing 15 studies found rates ranging from 1.4% to 70%, with higher occurrences typically observed in subgroups of individuals with advanced dementia or recent hospitalizations [3].

While evidence from other healthcare settings also supports geographic differences in delirium epidemiology, with Western countries reporting lower delirium detection rates compared to non-Western regions [10], cross-continental data within LTC settings are still lacking. Still, delirium in this context is widely recognized as globally underdiagnosed [11], partly due to its fluctuating nature, subtle clinical presentation [12], and frequent coexistence with dementia [13], which further complicates its assessment.

The aim of this study is to explore the epidemiology of delirium in LCT facilities (LCTFs) across multiple countries, focusing on point prevalence and assessment methods. This will serve as a foundation for a comprehensive understanding of the burden of delirium in LTC worldwide, offering valuable insights to inform awareness and address the challenges associated with its detection.

## Methods

The present study is a subanalysis of an international prevalence survey conducted on World Delirium Awareness Day (WDAD) on March 15th, 2023. The study was registered in the German Clinical Trials Register (DRKS00030002) and received ethical approval in all participating countries. The questionnaire comprised 39 questions and was administered online via SurveyMonkey [14]. All participating clinicians provided informed consent for the research at the outset of the questionnaire. Further details on study design, preparation, inclusion and exclusion criteria, and data collection procedures have been already described elsewhere [15, 16].

### Sample characteristics

Starting from 1664 completed surveys, we excluded those from emergency departments (n = 86), general wards (n = 758), acute care settings (e.g., high acuity, intermediate care, intensive care units; n = 649), and unspecified settings (n = 77), obtaining a final sample of 94 surveys from LTCFs.

### Delirium assessment

The survey respondents were asked not to directly evaluate delirium but to report the methods used for its assessment, the total number of residents in the ward/unit at each time point, and the number of residents identified with or without delirium by the ward/unit staff. Delirium point-prevalence was calculated by dividing the number of residents diagnosed with delirium by the total number of residents assessed for delirium at both 8 a.m. and 8 p.m.

Valid delirium detection tools were defined as measures recognized in the literature as reliable and validated [17]. The survey did not differentiate between screening and assessment for delirium as tools like the 4AT can be used for routine screening (e.g. all new residents) and for assessment based on suspicion of delirium (e.g. patient has a fall).

### Statistical analysis

Categorical data are presented as frequency (n) and percentages (%). Comparisons based on the type of setting were conducted using the Chi-square tests or the Fisher's exact test to explore differences between groups. Given the exploratory nature of the study, no adjustment for multiple comparisons was made. A two-tail p-value < 0.05 was set as statistically significant. The analysis was performed with R software, version 4.2.3.

## Results

**Fig. 1:**
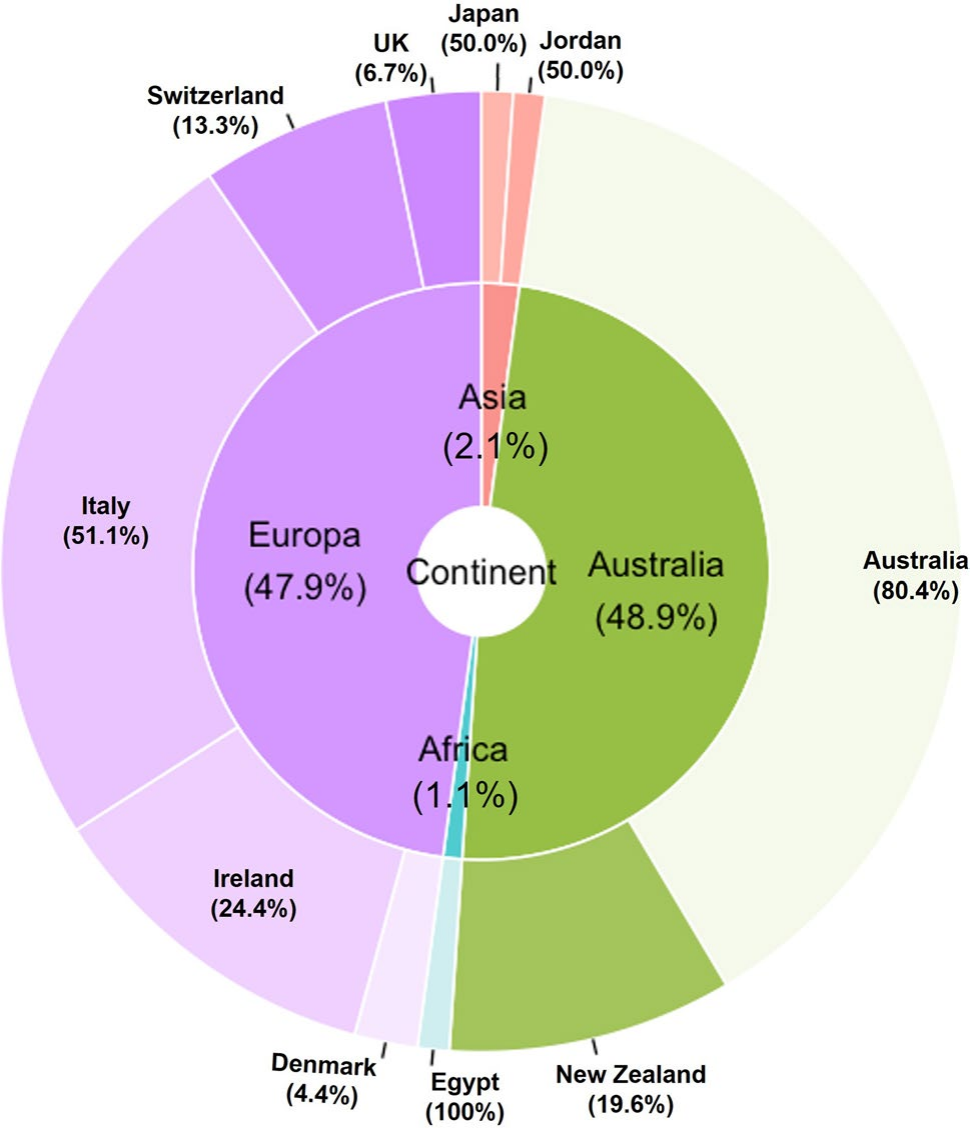
Distribution of survey participants across continents and countries

Figure 1 shows the distribution of the participating LTCFs across continents and countries. Most of the facilities were located in Europe and Australia (47.9% and 48.9%, respectively), with only a small proportion from Asia and Africa. No LTCFs from North or South America participated in the survey.

Out of ninety-four LTCFs, 65 (69.1%) were rehabilitation facilities (RFs) and 29 (30.9%) were nursing homes (NHs). The characteristics of the survey’s participants and LTCFs are presented in Table 1. Notably, in approximately 70% of the units/wards, residents were 75 years or above.

**Table 1:** Characteristics of survey participants: overall and by LTC setting (Rehabilitation vs. Nursing Home)

	Overall *n* = 94	Rehabilitation *n* = 65 (69.1%)	Nursing home *n* = 29 (30.9%)	*p*-value
Survey´s respondents (%)				0.703
Nurse	44 (46.8)	32 (49.2)	12 (41.4)	
Physician	31 (33.0)	20 (30.8)	11 (37.9)	
Other	17 (18.1)	11 (16.9)	6 (20.7)	
Patients age (%)				**0.021**
0–17 years	1 (1.1)	1 (1.5)	–	
18–75 years	19 (20.2)	17 (26.2)	2 (6.9)	
> 75 years	63 (67.0)	37 (56.9)	26 (89.7)	
Mixed	11 (11.7)	10 (15.4)	1 (3.4)	
Number of beds in LTCFs (%)				**0.001**
< 500	67 (71.3)	39 (60.0)	28 (96.6)	
500–1000	23 (24.5)	22 (33.8)	1 (3.4)	
> 1000	4 (4.3)	4 (6.2)	–	

Missing values for rehabilitation: profession = 2. Bold font indicates statistical significance

### Delirium assessment and prevalence

Overall, 12.4% (n = 221/1787) of residents were reported to have delirium in the morning, and 12.0% (n = 167/1389) were reported to have delirium in the evening. Notably, delirium point prevalence was significantly higher both in the morning (13.6% vs. 9.9%, *p* = *0.026*) and in the evening (14.1% vs. 3.3%, *p* < *0.001*) in RFs compared to NHs.

Information on delirium assessment procedures is shown in Table 2. Sixty-six (70.2%) LTCFs used a validated tool to detect delirium, with no significant differences between RFs and NHs (70.8% vs. 69.0%, respectively). Among the twenty validated delirium detection tools available, only five were selected: the 4AT (48.9%), the CAM (13.8%), the DSM-V criteria (4.3%), the 3DCAM (1.1%), and the NU-DESC (1.1%). The 4AT was the most frequently used tool both in RFs (52.3%) and NHs (41.4%), followed by CAM (15.4%) and personal judgment (20.7%) for RFs and NHs, respectively.

**Table 2:** Delirium assessment: overall and by LTC setting (Rehabilitation vs. Nursing Home)

	Overall *n* = 94	Rehabilitation *n* = 65 (69.1%)	Nursing home *n* = 29 (30.9%)	*p*-value
Type of delirium assessment used*				0.053
Personal judgment	13 (13.8)	7 (10.8)	6 (20.7)	
4AT	46 (48.9)	34 (52.3)	12 (41.4)	
CAM	13 (13.8)	10 (15.4)	3 (10.3)	
3DCAM	1 (1.1)	1 (1.5)	–	
DSM-V criteria	4 (4.3)	–	4 (13.8)	
NU-DESC	1 (1.1)	1 (1.5)	–	
Psychiatric consult	1 (1.1)	–	1 (3.4)	
None	2 (2.1)	1 (1.5)	1 (3.4)	
Other	11 (11.7)	9 (13.8)	2 (6.9)	
Validated delirium detection tool	66 (70.2)	46 (70.8)	20 (69.0)	0.879
Delirium assessment frequency				** < 0.001**
Once per day (24h)	15 (16.0)	13 (20.0)	2 (6.9)	
Twice per day (24h)	1 (1.1)	1 (1.5)	–	
Thrice per day (24h)	10 (10.6)	10 (15.4)	–	
More than thrice per day (24h)	2 (2.1)	2 (3.1)	–	
Only at admission	6 (6.4)	5 (7.7)	1 (3.4)	
Only in case of sudden changes of consciousness	40 (42.6)	17 (26.2)	23 (79.3)	
Other	16 (17.0)	14 (21.5)	2 (6.9)	
Profession primarily responsible for delirium assessment				** < 0.001**
Nurse	48 (51.1)	39 (60.0)	9 (31.0)	
Physician	24 (25.6)	7 (10.8)	17 (58.5)	
Specific delirium team	–	–	–	
Mixed professions	11 (11.7)	11 (16.9)	–	
None	6 (6.4)	3 (4.6)	3 (10.3)	
Other	1 (1.1)	1 (1.5)	–	
Delirium-awareness interventions				
At least one educational training about delirium	59 (62.8)	38 (58.5)	21 (72.4)	0.288
Delirium flyer for the staff	36 (38.3)	16 (24.6)	20 (69.0)	** < 0.001**
Delirium is mentioned in handovers	50 (53.2)	41 (63.1)	9 (31.0)	**0.008**
Pocket-cards for delirium assessment/management	9 (9.6)	9 (13.8)	–	0.084
Informational Posters about delirium	41 (43.6)	23 (35.4)	18 (62.1)	**0.029**
Delirium experts, known by the team and dedicated	31 (33.0)	15 (23.1)	16 (55.2)	**0.005**
Communication of delirium screening rate on unit/ward	30 (31.9)	27 (41.5)	3 (10.3)	**0.006**
None	7 (7.4)	4 (6.2)	3 (10.3)	0.772
Other	8 (8.5)	7 (10.8)	1 (3.4)	0.438

Missing values for rehabilitation: Type of delirium detection tool used = 2; Validated delirium detection tool = 2; Delirium assessment frequency = 3; Profession primarily responsible for delirium assessment = 4

Missing values for nursing home: Delirium assessment frequency = 1

Bold font indicates statistical significance

*Other available validated delirium assessment tools that weren’t selected by survey participants are: bCAM, CAM-ICU, CAMICU-7, DTS, DSM-IV criteria, DSM-VI criteria, ICDSC, SQiD, UB2, PAED scale, CAP-D, SOS-PD, pCAM-ICU, psCAM-ICU, sspCAM-ICU

**Fig. 2:**
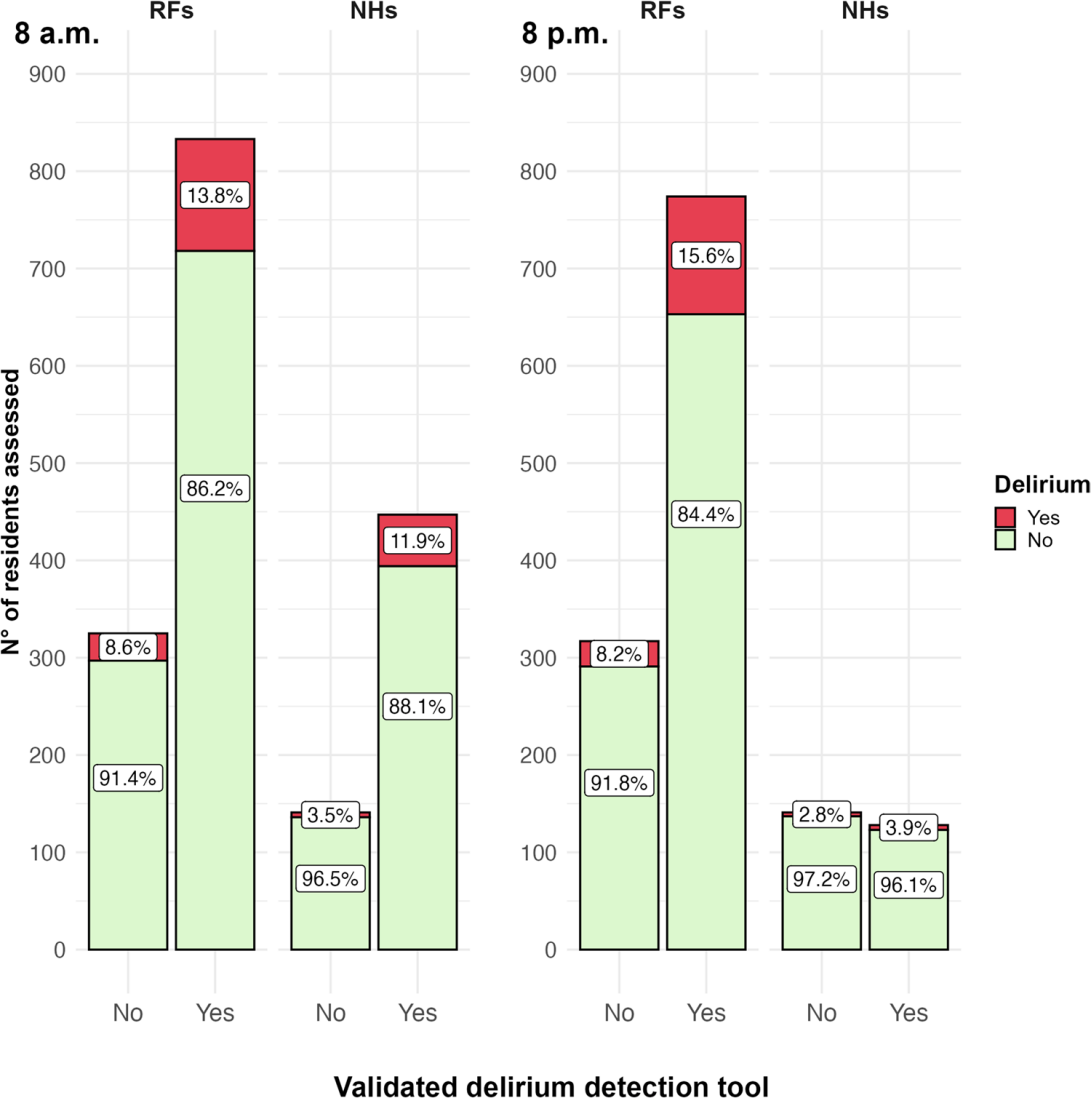
Delirium prevalence by LTC setting and the use of validated delirium tools

Figure 2 shows the delirium point prevalence in the two LTC settings based on the use of a validated delirium detection tool in the morning and the evening. A higher prevalence of delirium was detected at both time points among residents assessed with a validated tool both in RFs and NHs.

**Fig. 3:**
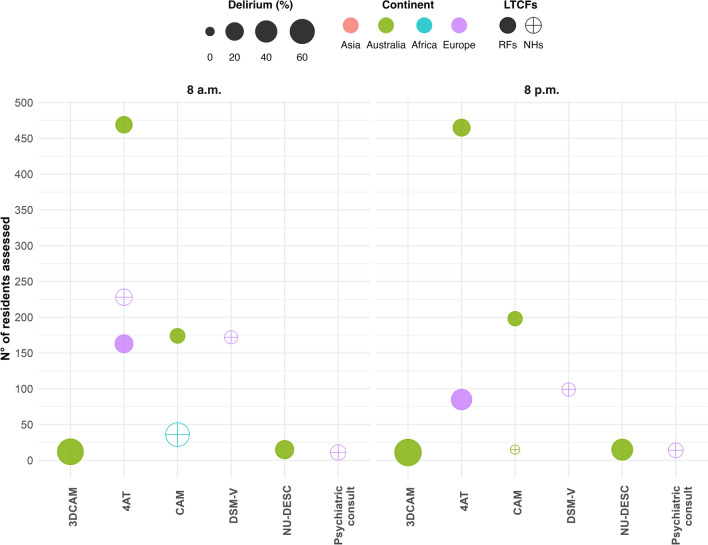
Delirium prevalence across continents and LTC settings by validated delirium detection tool

Delirium prevalence data at the two time points were further analyzed by considering only validated tools, highlighting differences across continents and LTC settings, as shown in Fig. 3.

RFs and NHs exhibited significant differences in the frequency of delirium assessment (Table 2). Most of the RFs declared to investigate it at least once a day (40.0% vs 6.9% in NHs), while the majority of the NHs only in case of sudden consciousness changes (79.3% vs 26.2% in RFs). Additionally, a significant difference emerged in the professional primarily responsible for delirium assessment, which was predominantly a nurse in RFs (60.0% vs. 31.0% in NHs) and a physician in NHs (58.5% vs. 10.8% in RFs).

Significant differences between RFs and NHs also emerged in the delirium-awareness interventions, as shown in Table 2. NHs reported a higher presence of delirium flyers for the staff (69.0% vs 24.6% in RFs, p < 0.001), informational posters (62.1% vs. 35.4% in RFs, p = 0.029), and delirium experts (55.2% vs. 23.1% in RFs, p = 0.005). Conversely, RFs reported more frequent communication about delirium in handovers (63.1% vs. 31.0% in NHs, p = 0.008) and delirium screening rates on unit/ward (41.5% vs. 10.3% in NHs, p = 0.006).

## Discussion

Our study found an overall reported delirium point prevalence of roughly 12%, with higher rates in RFs compared to NHs. Approximately 70% of the participating LTCFs reported using a validated detection tool, with the 4AT being the most commonly employed. Notably, most RFs reported daily delirium assessment, primarily carried out by nurses, whereas most NHs assessed delirium only in case of changes in consciousness, with the evaluation predominantly performed by physicians.

To date, most research on delirium has primarily focused on acute care settings [10, 18, 19]. Studies examining the occurrence of delirium in LTCFs have reported varying rates, influenced by the characteristics of the population and the detection tools employed. Our findings contribute to this underexplored area and highlight that the use of validated instruments enhances the detection of delirium.

The overall prevalence of delirium in RFs has been reported to range from approximately 10% to 16% [20]. A recent multicentric study investigating delirium prevalence in older patients admitted to RFs identified a prevalence of 18% using the 4AT screening tool [21], which aligns with the prevalence observed in RFs in our study when validated screening tools were employed. In contrast, a narrative review highlighted a wide range of delirium prevalence in NHs, varying from 1.4% to 70.3% [22]. This considerable variability likely reflects differences in detection tools, diagnostic criteria, and population characteristics, including the prevalence of conditions predisposing to delirium [23]. For instance, a nationwide multicentric point-prevalence study conducted across 71 NHs reported a delirium prevalence of 36.8% detected using the 4AT, with functional dependence, malnutrition, and dementia identified as significant risk factors [24]. Although the prevalence of delirium observed in NHs in our study falls within the range reported in the literature, it is at the lower end of that spectrum. This could be attributed to differences in the frequency of delirium assessment, the tool used, the clinical profile of the population, as well as structural differences in NHs across countries.

Furthermore, the difference in prevalence rates between RFs and NHs observed in our study is notable, with RFs reporting a higher prevalence of delirium at both time points. Several factors may account for this disparity. One potential explanation is the frequency of delirium assessment, which was reported to occur daily in most RFs, while only in response to a sudden change in consciousness in NHs. This difference in routine assessment practices likely contributed to the higher detection rates observed in RFs. Residents’ characteristics may have also played a role in the observed disparity, although the survey design did not permit an more-in-depth exploration of these factors. Another contributing factor could be the type and availability of healthcare personnel responsible for conducting delirium screening. In RFs, assessment was primarily carried out by nurses, who usually are present 24 h a day, allowing for consistent monitoring. In contrast, NHs often relied on physicians for this task, but their availability is typically limited to a few hours per day. This difference in personnel availability and roles may have influenced delirium detection rates and could also explain the variability in prevalence observed in NHs across the two time points.

Further consideration must be given to the selection of tools and the expertise of personnel involved in delirium assessment. Many delirium detection tools require specific training to ensure accurate use, and variations in training levels could influence the reliability of detection [11]. Additionally, several medical conditions, such as dementia or aphasia, can impact assessment accuracy, potentially resulting in the over- or under-estimation of delirium prevalence depending on the tool used [13]. Lastly, some instruments are only validated for screening and require a diagnostic interview for confirmation. Consistent with previous research in different settings, our study highlights significant variability in reported delirium positive score rates based on the choice of detection tools and the timing and frequency of evaluations [25].

Together, these factors underscore the complex interplay between screening practices, resident demographics, clinical profile, and healthcare personnel expertise in shaping delirium prevalence across various care settings and countries.

This survey did not investigate whether clinicians involve family members or volunteers to enhance delirium detection, especially in the NH settings. Previous studies in acute care settings suggest that family members may play a significant role in the early recognition of delirium, as they are often able to detect subtle cognitive changes earlier than healthcare staff [26–29]. Several tools have been developed to assist families in delirium detection, including the Family Confusion Assessment Method (FAM-CAM), the Sour Seven Delirium Questionnaire (Sour Seven), and the Informant Assessment of Geriatric Delirium (I-AGeD). While these tools can support the identification of delirium, their complexity and the time required for their application may pose challenges in practice. An alternative approach is the Single Question in Delirium (SQiD), which involves asking a simple yes/no question, “*Do you think [name of patient] has been more confused lately?”* to the family and carers. This tool has demonstrated good sensibility and specificity in detecting delirium [30]. Engaging family members, particularly in NH settings, may provide valuable support to healthcare providers in the early detection and timely management of delirium.

Future approaches to delirium detection in LTCFs could also benefit from exploring the integration of emerging technologies, such as wearable devices and in-room sensors. These devices, which have already shown promise in monitoring age-related issues among this resident population [31–33], hold significant potential to improve the early detection and management of delirium, while also alleviating staff workload [34]. Still, further research is needed to validate their applicability and effectiveness in this field.

This study has several limitations. First, clinicians and researchers with a specific interest in delirium may have been more inclined to participate in the WDAD survey, potentially introducing selection bias. Moreover, some participants may have belonged to different units within the same institution. Consequently, the findings may not fully reflect the overall LTC setting across different countries and continents. Moreover, the low participation rate of LTCFs from Asia and Africa, along with the lack of data from American LTCFs, further restricts the generalizability of our findings. Second, the lack of a direct delirium assessment requires careful consideration. Lastly, we were unable to verify the data collection or entry methods employed by each LTCF.

Future surveys should aim to address these limitations by actively recruiting clinicians who are less frequently involved in delirium-related research and by overcoming barriers to enhance the participation of LTCFs from Africa, America, and Asia, thereby highlighting potential epidemiological and management differences.

## Conclusion

Despite the heightened risk of delirium among individuals in LTCFs driven by factors such as functional impairments, multiple comorbidities, polypharmacy, and cognitive decline, its detection in these settings remains suboptimal. The LTC setting, particularly across different countries, presents a diverse and heterogeneous landscape characterized by variations in resident and healthcare personnel characteristics. This heterogeneity contributes to inconsistencies in clinical practices for delirium detection and management and underscores the need for greater efforts toward a more standardized and objective characterization of this condition in LTCFs worldwide.

## Supplementary Information

Below is the link to the electronic supplementary material.

Supplementary file1 (DOCX 28 KB)

## Data Availability

Data are available in anonymous form upon reasonable request addressed to the corresponding author.
