# Supplementary material for: Epidemiology and assessments of delirium in nursing homes and rehabilitation facilities: a cross-country perspective
Source: Eur Geriatr Med. 2025 Apr 28;16(6):1909–17. doi: 10.1007/s41999-025-01207-x (PMC12743668; doi:10.1007/s41999-025-01207-x)
Supplement: Supplementary file 1 — Supplementary file1 (DOCX 28 KB) [file 41999_2025_1207_MOESM1_ESM.docx]

**EPIDEMIOLOGY AND ASSESSMENTS OF DELIRIUM IN NURSING HOMES AND REHABILITATION FACILITIES: A CROSS-COUNTRY PERSPECTIVE**

Alice M. Ornago^1,2^, Elena Pinardi^1,2^, Maria Cristina Ferrara^1^, Suzanne Timmons^3^, Chukwuma Okoye^1,2,4^, Alberto Finazzi^1^, Paolo Mazzola^1,4^, Peter Nydahl^5,6^, Rebecca von Haken^7^, Heidi Lindroth^8,9^, Keibun Liu^10^, Alessandro Morandi^11,12^, Giuseppe Bellelli^1,4^ on behalf of the *WDAD Study Group*

**Affiliations:**

1. School of Medicine and Surgery, University of Milano-Bicocca, Milan, Italy
2. Aging Research Center, Department of Neurobiology, Care Sciences and Society, Karolinska Institutet and Stockholm University, Stockholm, Sweden
3. Mercy University Hospital and St Finbarr's Hospital, Cork, Ireland
4. Acute Geriatric Unit, IRCCS San Gerardo Foundation, Monza, Italy
5. Nursing Research, University Hospital Schleswig-Holstein, Kiel, Germany
6. Institute of Nursing Science and Development, Paracelsus Medical University, Salzburg, Austria
7. Department of Surgery, University Hospital Mannheim, Mannheim, Germany
8. Division of Nursing Research, Department of Nursing, Mayo Clinic, Rochester, MN, USA
9. Center for Aging Research, Regenstrief Institute, Center for Health Innovation and Implementation Science, School of Medicine, Indiana University, Indianapolis, IN, USA
10. ICU Collaboration Network (ICON), Tokyo, Japan
11. Intermediate Care and Rehabilitation, Azienda Speciale Cremona Solidale, Cremona, Italy
12. University of Brescia, Brescia, Italy

A. M. Ornago and E. Pinardi are co-first author.

**KEYWORDS:** delirium; long-term care; nursing homes; rehabilitation; epidemiology

**RUNNING TITLE:** Epidemiology of delirium in nursing homes and rehabilitation facilities

**Corresponding author:**

Elena Pinardi

School of Medicine and Surgery, University of Milano-Bicocca

Piazza dell’Ateneo Nuovo 1, Milan, Italy

e-mail address: [e.pinardi@campus.unimib.it](mailto:e.pinardi@campus.unimib.it)

ORCID: orcid.org/0009-0004-8068-9406

**Acknowledgements:**

- **Appendix 1a:** Acknowledgement of collaborative authors (WDAD Study Group).
- **Appendix 1b:** Acknowledgement of participating clinicians.

**Appendix 1a: Acknowledgement of collaborative authors.**

The “WDAD Study Team” comprises of 159 collaborative authors:

Ahmed Gaber Ahmed Abdalgany, MBBCH., Sarah Magdy Abdelmohsen, M.D., Amjad Aburaas, M.D., Christina Aggar, M.D., Bashir Abobaker Albakosh, M.D., Hamza Ismail Ahmad Alhamdan, M.D., Akram Waled Rajab M Aljbali, M.D., Farah Alkandari, M.D., Akram Alkaseek, MBBCh, MRCS, Daniel Anders, R.N., Marsali Anderson, M.B. Ch.B. B.Sc., Shelly Ashkenazy, Ph.D., Thiago Avelino, Kasia Siobhan Bail, Ashraf Bakri, M.D., Alaa Mohamed Ali Baroum, Bronagh Blackwood, Ph.D., Jennifer Brendt-Müller, M.Sc., Angelika Brobeil, B.B.A., Richard Burke, M.D., Tru Byrnes, D.N.P., Stefano Cacciatore, M.D., Maria Cahill, MSc, Maria Ana Canelas, Ida Carroll, M.Sc., Amy Conley, Mb.Ch.B., Maria Costello, M.D., Shannon Cotton, R.N., Mandy Couser, B.Sc., Ana Rita Cunha Salgado, M.D., Elizabeth Cusworth, M.S., Vera Cvoro, M.D., Jordanna Deosaran, M.D., Merel Diebels, M.Sc., Elaine A Docherty, M.Hp., Vera von Dossow, M.D., Megan Drennan, M.D., Akram Amin Egdeer, MBBCH., Patrick Eichelsheim, MANP., Aissha Ali Saleh Elagili, M.D., Saifaleslam Jamal Elsahl, M.D., Hajer Alsadeg Mohammed Elshaikh, M.D., Christina Emme, Ph.D., Matthias Thomas Exl, M.Sc., Azza Fathi, M.D., Melanie Feige, Dipl. Paed., Kirsten Fiest, Ph.D., Marleta Irene Joy Fong, Ms.N., Mikita Fuchita, M.D., Carol Gaffney, M.D., Carola Gimenez-Esparza Vich, M.D., Nicole Feldmann, M.Sc., Rachel Fitzgerald, M.D., Neasa Fitzpatrick, M.D., Marleta Irene Joy Fong, M.N. C.N., Maria Adela Goldberg, M.D., Nienke Golüke, M.D., Mirjam de Graaf, MSc, Engelina Groenewald, M.D., Renate Gross, M.D., Camilla Grube Segers, M.D., Renate Hadi, R.N., Qusai Ahmad Hasan Hamdan, B.S., Bahaeddin Ben Hamida, MBBCH., Mohamed Hassan Hamza. M.D., Breanna Hetland, Ph.D., Jane Adele Hopkins, M.HA., John Hopkins, MBChB., FRCPsych., Sarah Ahmed Atef Mohamed Ibrahim, MBBCH., Guglielmo Imbriaco RN MSN, on behalf of Aniarti, Italian Association of Critical Care Nurses, Azienda USL di Bologna Italy, Shigeaki Inoue, Ph.D., Arveen Jeyaseelan, M.D., Ali Jawad Kadhim, MBChB., Sabrina Kohler, MBBS. FRACP., Rens Kooken, M.D., Anna St. Korompeli, Ph.D., Lars Krüger, M.Sc., Ayman Salim Abu Khutwah, Puck de Lange, M.D., Sharon Liefrink, R.N., Yu-min Lin, M.D., Shi Pei Loo, M.D., Sara Beatriz Lopes Rodrigues, M.D., Allan MacDonald, M.D., Gillian Madders, M.D., Claudia Massaro, M.D., Kerri Maya, M.SL., Sofia Manioudaki, M.D., Natalie McAndrew, P.hD., Stewart McKenna, M.D., Isabel Maria Metelo Coimbra, M.D., Teresa Miranda, M.D., Mohamed Anwar Abdelsalam Mohamed, M.D., Mushin Mohammed Elhadi Agbna Mohammed, M.D., Malissa Mulkey, Ph.D., Fariha Naeem, M.D., Kensuke Nakamura, Ph.D., Mi-Ryeo Nam, Elaine Newman, MSc, Renae Nicol, CNC., Claire Noonan, Msc. A.P., Maria Inês Nunes Oliveira Lopes, M.D., Zina Otmani, Cynthia Olotu, M.D., Alice Margherita Ornago, M.D., Susan O’Reilly, M.Sc., Valerie Ozorio, M.D., Jessica Palakashappa, M.D., Tej Pandya, M.D., Panagiota Papadea, M.D., Metaxia Papanikolaou, M.D., Rose S. Penfold, M.PH., Elena Pinardi, M.D., Inês Filipa Pinto Pereira, M.D., Chirantha Premathilaka, M.D., Monica Pop-Purceleanu, M.D., Marlene Puchegger, B.Sc., Nouralddeen Mohammed Qalhoud, M.D., Terence J Quinn, M.D., Maike Raasing, Ph.D., Dalia Talaat Ragheb, M.D., Prasad Rajhans, M.D., Nuri Ramadan, M.D., Mushabbir Hossain Rubel, M.D., Kate Sainsbury, B.Sc., Francesco Salis, M.D., Florian Schimböck, M.Sc. Med, Roman Schmädig, M.Sc., Yvonne Schoon, M.D., Deepak Sethia, M.D., Edith Sextl, M.Sc., Bhagyesh Shah, M.D., Dua’a Shaout, M.D., Alaa Fouad Sharabi, M.D., Lynn Shields, M.D., Kendall Smith, M.D., Linda Smulders-van Dam, MSc, Roy L Soiza, M.D., Andrea Spiegler, M.Sc., Lucy Stocks, MBBS., Stefan Sumerauer, M.Sc., Stephanie Tam, B.Sc., Aik Haw Tan, MBChB., FRACP., Suzanne Timmons, Ph.D., Peter Tohsche, M.Sc.N., Raquel Gouveia Torres, M.D., Chantal Toth, D.N.P., Vasiliki Tsolaki, Ph.D., Iben Tousgaard, M.PH., Roberta Esteves Vieira de Castro, Birgit Vogt, Ph.D., Erica Walsh, M.D., Kristel Ward-Stockham, MANP., Melinda Webb-St. Mart, M.PH., Franziska Wefer, M.Sc., Mariajne Elisabeth Wijnen-Meijer, Ph.D., Hilde Wøien, M.D., Inke Zastrow, M.Sc., Maria Beatrice Zazzara, MD

**Appendix 1b: Acknowledgement of participating clinicians**

The authors would like to gratefully acknowledge the following participating clinicians for supporting the 2023 WDAD Study Team and survey: Breel Montaser (**Afghanistan**); Mariana Ojeda; Ana Maria Mazzola; Ana Paula Cirese; Maria Eugenia Teran; Evangelina Pereyra Zamora; Giuliano Gaudenzi;Daniela Olmos; Sanchez Natalia Elizabeth; Mariela Mogadouro; Matias Cattoni; Maria Belen Frate; Monica Quinteros; Miguel Ángel Salas ;Carlos Nahuel Brito (**Argentina**); Barbara Cass; Donna Wheeldon; Emma Ham; Hannah Zhang; Isabelle Brewer; Kate Baptist; Kate Lipson; Myfanwy Guay; Nisha Kuzhuvelil; Sonia Hiam; (**Australia**); Mata Alexander; Patrik Heindl (**Austria**); Barbara Flynn; Brooke Blythe; Christine MacDonald; Dori-Ann Martin; Eric Sy; Eugene E. Mondor; Gabriela Costa; Jennifer Armstrong; Kari France; Katherine Kissel; Kevin Iwaasa; Kirsten Deemer; Natalia Jaworska; Oleksa Rewa; Sebastien Roulier; Shibi George; Susan Anderson; Tanya Mailhot; Trudy Eggleton (**Canada**); Fernando Tirapegui (**Chile**); Ann-Kirstine Hansen; Anne-Dorte Krapper; Anders Hudsen Madsen; Anne Højager Nielsen; Anschelilka Pedersen; Bianca Bech; Camilla Bekker; Charlotte Riis; Edel Laursen; Eva Laerkner; Janet Froulund Jensen; Karina Jakobsen; Kristine Mildahl Kjaergaard; Laura Krone Larsen; Lene Lehmkuhl; Lone Høilund Kristiansen; Linette Thorn; Marie Damgaard Winther , Maja Søndergaard Nielsen; Martin Schultz; Monica Baunbæk; Mette Gryholt Fenger; Anna Palmen; Pernille Reck Miller; Rikke Salomon; Sally Jakobsen; Sanne Thers Lauritzen; Susanne Fischer; Tina Tang Fredenslund (**Denmark**); Dalia Talaat Ragheb; Ahmed Allam; Ahmed.H.Elmasry; Aiman Al-Touny; Aliae Mohamed Hussein; Ali Wael; Ali Aldarraji; Ahmed Abdelhalim; Bassam Fahmy; Dinah Nabil; Ahmed Abdelrahman; Rana Ahmed Adel; Hossam Mohamed Saad Ali; Islam Galal Sayed; Kyrillos Wassim; Mohamed Ahmed Adel (**Egypt**); Alexander Kaestner; Lisa Fink (**Germany**); Aggeliki Detta; Tsioka Agorista; Anna Efthymiou; Archontia Mahairidou; Artemis Vekrakou; Athanasiou Paraskevi; Christina Lymperatou; Christos Ntiakalis; Konstantinous Kyriakoulis; Eftychia Kourtelessi; Eleni Spiropoulou; Evangelia Michail Michailidou; Evi Tsigou; Gkika Dimitra; Georgios Papathanakos; Ioannis Andrianopoulos; Aikaterini Sakagianni; Aikaterini Katsogianni; Theodora Katsarou; Maria Bolaki; Maria Laskou; Maria Gianiki; Iliana Tryfonidou;Lampros Banos;Maria Panoutsopoulou; Anastasia Zagorianou; Niki Rouvali; Nektaria Xirouchaki; Panagiotis Poulikakos;Eirini Papagiorgiou; Mantzafleri Peristera Eleni; Mpoutzouka Eleni; Stiliani Andreadou; Kyriaki Kolovou; Vasiliki Karaouli; Panagiota Stamou; Stavros Mantzoukis; Theodora Soulele; Athanasia Tsirogianni; Theodoros Aslanidis; Theofilia Theodoridou; Apostolos Bakas; Myrto Tzimou; Vasiliki Chantziara; Zisopoulou Vasiliki (**Greece**); Ghanshyam Yadav; Abhishek Kumar; Amit Goel; Arun Sharma; Biswanath Sahoo; Anil Kumar; Manish Gupta; Meraj Ahmad; Nikhil Kothari; Trishita Saha; Garima Mishra; Gaurav Pandey; Jacob George; Kalai Selvan; Lalit Singh; Mohan Gurjar; Madhusudan Kalluraya; Syed Nabeel Muzaffar; Prashant Mohan; Ritu Singh; Rupali Patnaik; Shiv Shanker Tripathi; Vijay Sundarsingh; Vipin Kumar Singh (**India**); Hussein Al-Najjar; Istevan Khazmi (**Iraq**); Ann Prendergast; Ann Sheehan; Anna Doherty Walsh; Anne-Marie Daly; AnnJane Kelly; Caoimhe McManus; Catherine Condon;Diane Kell; Diarmaid Semple, Lousie Owens, Tara Connaughton, Katie Morris; Michael Dowling; Edel Byrne; Eleanor Morris; Hafsah Ali; Siofra Hearne; Leona Bannon; Louise Kelly; Margaret Sheehan Velthuysen; Natalie McEvoy; Niamh Annmarie O'Regan; Oisin Hannigan; Olive Vereker; Patrick Doyle; Paul Claffey; Paul McElwaine; Colm Ryan; Suzanne Laffan (**Ireland**) Miriam Abuhazira (**Israel**); Clara Agostino; Yanely Sarduy Alonso; Ilaria Bandera; Enrico Brunetti; Luca Bucciarelli; Monica Cadei; Antonio Ciambrone; Alessandro Di Risio; Rosa Filipelli; Luigi Francioni; Alessandro Galazzi; Barbara Gamba; Giordana Gava; Simona Gentile; Emma Giovannini; Angela Iurlaro; Maria Legierska; Alessandro Monesi; Giulia Principato; Daniela Quattrocchi; Daniela Perelli Ercolini; Sabina Perelli Ercolini; Daniela Petronela Radeanu; Carla Recupero; Alessandro Reggiani; Antonella Risoli; Barbara Romagnoli; Loretta Ruggeri; Elena Trotta; Salvatore Tupputi; Edoardo Varatta; Valentina Viani (**Italy**); Hidenori Kasuya; Ayaka Sakamoto; Daisuke Kawakami; Haruki Ishizuki; Masaru Matsumoto; Go Haraguchi Sakakibara; Hayami Hajime; Shigeaki Inoue; Kawabata Jun; Koji Yamashita; Kenzo Ishii; Maiko Nakamura; Kensuke Nakamura;Tomomi Furumaya; Nobuo Sato; Shinichiro Ohshimo; Takero Terayama; Takumi Nagao; HIidenori Sumita ;Yoshitaka Aoki; Yuuki Ozaki (**Japan**); Aya Omar Mohammad Alaqtash; Joud Mwaffq Hussein Sharadga; Ahmad Omar Abu Hamideh; Anas Aljaiuossi; Saleh Ali Mohammed Ba Shammakh; Dima Rahhal; Hasan Mohammad Haj Freej Husam Emad Ibrahim Matalqah; Mohammad Emad Moh'd Abu Hussein; Dana Jehad Samardali; Razan Waleed Yaseen (**Jordan**); Abdullah Tarboush (**Kuwait**); Hiba Hamdar (**Lebanon**); Akram Alkaseek; Ayman Abu Khutwah; Abdualmalek Algomaty; Abdulrhaman Jaber; Ahmed Mohamed Alsaedi; Aisha Bojazyah; Amani Mohammed Senousi Shehab: Asma Moftah; Aya Fageir; Ali Kredan; Fairouz Mohammed Mustafa Alghadamisi; Fatoom Alowjali; Tasneem Mohammed Abu Bakr Ali; Hajar Alkokhiya Aldare;Hamida El Magrahi; Heba Khalifa Mohammed; Hibah Bileid Bakeer; Mabroukah Omar; Marwa Morgom; Mohammad Yahmad; Rabab Mohammed Salim Alkurghali; Saifaleslam Elsahli; Sarah Alfaqaih; Talat Ahmed Abu Salem; Zaynab Omar Aldayri (**Libya**); Luis Antonio Gorordo-Delsol; Karen Selene Rivera Martínez (**Mexico**); Linda Smulders; Mirjam de Graaf; Nienke Golüke; Puck de Lange (**Netherlands**); Himali Aickin; Jane Walton; Lijo John Tharakan; Melania Tele; Richard Worrall **(New Zealand**); Anita Dahl; Antonija Petosic; Bjørn Erik Neerland; Brita Fosser Olsen; Hans Frank Strietzel; Inga Akeren; Kristin Naustdal; Marja Wanne Hoff; Mette Dokken; Mona Morland; Rutt Katrine Bollingmo; Renate Ramfjord; Silje Bådsvik; Stian Søllesvik Qvam;Trine Sortland Triumf (**Norway**); Moustafa Kotb Elmala (**Oman**); Luis Daniel Umezawa Makikado (**Peru**); Margarida Ferreira Sousa; Ana Filipa Pires; Antero do Vale Fernandes; Carla Araújo Costa; Carla Margarida Coelho Marques Abrantes Teixeira Claro da Fonseca; Ana Maria Pina de Albuquerque; Liliana Filipa Pires Pereira (**Portugal**); Chul Park; Dae-sang Lee; Sua Jo; Uiwon Ko; Chi-Min Park; Ire Heo; Tae Wan Kim; Hohyung Jung; Byunghyuk Yu; Hak-Jae Lee; Jae Kyeom Sim; Donghoon Kim; Song-I Lee; Sua Kim (**Korea**); Águeda Ojados Muñoz; Ana Maria Del Saz Ortiz;Ana María Prieto de Lamo; Ana Pardo; AsuncionVergara Sanchez; Beatriz de Ramon Rodriguez; Beatriz Oliver Hurtado; Cándido Pardo Rey; Concepcion Ruiz Iniesta; Esther Aguirre Recio; Federico Minaya González; Jennifer Carolina Figueroa Falconí; Felix Martin Gonzalez; Francisco Luis Pérez Caballero; Sofia Garcia-Manzanedo; Gina Rognoni Amrein; Gemma Rialp; Inmaculada Georgia Garcia Gomez; Jesus Caballero; Jesus Priego; Jose Luis Perez Vela; Josep Trenado Álvarez; Lorenzo Lopez; Lorena-Zoila Peiró Ferrando; Luis J. Yuste Domínguez; Manuela Garcia Sanchez; Maria Riera-Sagrera; Marina Orantes Pozo; Eulalia; Romero Granados; Maria Luisa Navarrete Rebollo; Maria Cruz Martin; Elena Martínez Quintana; Miguel A. Gonzalez Gallego; Monica Garcia Simon; Mercedes Ibarz; Mireya Molina Cortés; Gabriel Heras La Calle; Sol Fernández Gonzalo; Marta Yagüe-Huertas; Nuria Martinez Sanz; Francisca Pino Sánchez; Hipolito Perez Molto; Eva Benveniste Perez; Paula Rodriguez Pedreira; Paolo Cardenas Campos; Maria del Pilar Eugenio Robaina; Jose M Gomez; Susana Temprano Vazquez; Tomas Munoz-Martinez (**Spain**); Eliana Almasri; Shahd Adnan Alhindi (State of Palestine); Abdalelah Salah Mohammed Hussein; Ibrahim Adel; Abdalsalam Saleh Mohammed Mussa; Essam Eldien Abuobaida; Ibrahim Mutwakil Gamal Ahmed; Lina Sameer; Mariam Alazraa Mohamed; Rashad Abdallah Esmaeel; Mohammed Faroug Abdulmajeed Al-awadh; Nidhal Isameldin Abdalla Siddig; Osama Gamal Nubi; Watfaa Siddeg (**Sudan**);Yvonne Kröger; Agnieszka Wysocka; Ana Patricia da Silva Morais; Andrea Gagliardi; Arnaud Forestier; Bettina Foord; Bettina Vessaz; Celine Lomme; Christian Emsden; Esther Naef; Lilith Stalder;Luca Cioccari; Nadja Annen;Natalie Leuenberger; Pascale Schaub; Regula Pfäffli Scheurer; Stephan Dinkler; Samanta Septinus; Sandra Haubner; Sheila Matti; Sibylle Fischbacher; VIvianne Chanez; Yvonne Seiffert (**Switzerland**); Hala Bakro; Sami Sannoufa (**Syria**); Sana Landolsi (**Tunesia**); Monzir Mohamedelmahdi Ali Salih (**UAE**); Alison McCulloch; Amy Conley; Andrew McCleary; Anne-Maree Robinson; Antonia Hargadon-Lowe; Cara Hammond; Carolyn Green; Claire Sturrock; Conor James McCann; Craig Stewart; Dawn Goodacre; Dervla Carroll; Diane Brisbane; Drew Crooks; Fahed Gareb; Effie Dearden; Eldilla Rizal; Elisabeth Sullivan; Ellie McIntosh; Emma Williamson; Felicity Baines; Fiona Tullo; James Yeado; Matthew Turner; Jamil Marhatta; Jasmine Hart; Jennifer Lonnen; Jiajin Kwong; Katie Murray; Katrina R. Bell; Kirsty Houston; Laura Tous Sampol; Laura Elizabeth Henderson; Lindsay Kenworthy; Martin Cowie;Mary Melville; Pam D'Silva; Rosie Cervera-Jackson; Rachael; Sarah Thomson; Sofia Cuevas-Asturias; Tabitha Brough; Victoria Richmond; Wan Norshuhada Wan Montil; Will Boatman; Zoe Spence (**United Kingdom**); Cynthia Bell; Christina Bohl; Dale M. Needham; Dianne Bettick; Evelyn Ivy Mwangi; Lillian Banchero; Whitney Smith (**United States**).
